# Impact of Vitamin D Supplementation on Influenza Vaccine Response and Immune Functions in Deficient Elderly Persons: A Randomized Placebo-Controlled Trial

**DOI:** 10.3389/fimmu.2019.00065

**Published:** 2019-02-08

**Authors:** Nicolas Goncalves-Mendes, Jérémie Talvas, Christian Dualé, Aline Guttmann, Violaine Corbin, Geoffroy Marceau, Vincent Sapin, Patrick Brachet, Bertrand Evrard, Henri Laurichesse, Marie-Paule Vasson

**Affiliations:** ^1^University of Clermont Auvergne, INRA, UMR 1019 Human Nutrition Unit, Clermont-Ferrand, France; ^2^CHU Clermont-Ferrand, INSERM CIC 1405, University of Clermont Auvergne, Clermont-Ferrand, France; ^3^CHU Clermont-Ferrand, Biostatistics Unit, University of Clermont Auvergne, Clermont-Ferrand, France; ^4^CHU Clermont-Ferrand, Infectious Diseases Department, University of Clermont Auvergne, Clermont-Ferrand, France; ^5^CHU Clermont-Ferrand, Biochemistry and Molecular Biology Department, University of Clermont Auvergne, Clermont-Ferrand, France; ^6^CHU Clermont-Ferrand, Immunology Department, University of Clermont Auvergne, Clermont-Ferrand, France; ^7^CHU Clermont-Ferrand, Nutrition Unit, University of Clermont Auvergne, Clermont-Ferrand, France; ^8^Centre Jean Perrin, Nutrition Unit, Clermont-Ferrand, France

**Keywords:** vitamin-D deficiency, influenza vaccination, aging, cathelicidin, cytokine, leukocyte phenotyping, randomized trial

## Abstract

**Background:** Immunosenescence contributes to reduced vaccine response in elderly persons, and is worsened by deficiencies in nutrients such as Vitamin (Vit-D). The immune system is a well-known target of Vit-D, which can both potentiate the innate immune response and inhibit the adaptive system, and so modulate vaccination response.

**Objective:** This randomized placebo-controlled double-blind trial investigated whether Vit-D supplementation in deficient elderly persons could improve influenza seroprotection and immune response.

**Design:** Deficient volunteers (Vit-D serum <30 ng/mL) were assigned (V1) to receive either 100,000 IU/15 days of cholecalciferol (D, *n* = 19), or a placebo (P, *n* = 19), over a 3 month period. Influenza vaccination was performed at the end of this period (V2), and the vaccine response was evaluated 28 days later (V3). At each visit, serum cathelicidin, immune response to vaccination, plasma cytokines, lymphocyte phenotyping, and phagocyte ROS production were assessed.

**Results:** Levels of serum 25-(OH)D increased after supplementation (D group, V1 vs. V2: 20.7 ± 5.7 vs. 44.3 ± 8.6 ng/mL, *p* < 0.001). No difference was observed for serum cathelicidin levels, antibody titers, and ROS production in D vs. P groups at V3. Lower plasma levels of TNFα (*p* = 0.040) and IL-6 (*p* = 0.046), and higher ones for TFGβ (*p* = 0.0028) were observed at V3. The Th1/Th2 ratio was lower in the D group at V2 (D: 0.12 ± 0.05 vs. P: 0.18 ± 0.05, *p* = 0.039).

**Conclusions:** Vit-D supplementation promotes a higher TGFβ plasma level in response to influenza vaccination without improving antibody production. This supplementation seems to direct the lymphocyte polarization toward a tolerogenic immune response. A deeper characterization of metabolic and molecular pathways of these observations will aid in the understanding of Vit-D's effects on cell-mediated immunity in aging. This clinical trial was registered at clinicaltrials.gov as NCT01893385.

## Introduction

Influenza infection occurs in people of all ages, but complications are more frequent in elderly persons (1, 2). This is partly due to immune dysfunctions caused by aging, i.e., immunosenescence, which can be explained by increased antigenic challenges and chronic inflammation, worsened by deficiencies in nutrients such as Vitamin D (Vit-D) (3–5). Vit-D deficiency, defined as 25-hydroxyvitamin D [25-(OH)D] serum levels below 30 ng/mL, is seen in 50–80% of the French population (6, 7). Vit-D deficiency occurs more frequently in older adults than in young ones because of their lower endogenous Vit-D synthesis, and because of their often reduced dietary intake (8).

Recent studies have demonstrated an expression of the Vit-D receptor (VDR) in almost all immune cells, suggesting that Vit-D has anti-infectious and immunomodulatory effects (9). These cells also express 1α-hydroxylase CYP27B1, which converts 25-(OH)D into bioactive 1,25-(OH)_2_D (10, 11). Among the mechanisms contributing to the anti-infectious properties of Vit-D, the production of antimicrobial peptides such as cathelicidin (also called LL-37) has been reported (12). Cathelicidin, an antimicrobial polypeptide produced by phagocyte cells, provides protection against bacterial infection. Its expression in respiratory epithelium is upregulated by active metabolites of vitamin D (12, 13). Moreover, cathelicidin has direct antiviral effects against influenza (14, 15). Tripathi et al. have partially characterized the mechanism of this activity based on the inhibition of viral replication at early stages of intracellular life cycle of the virus (16, 17). Recent findings show that cathelicidin is also able to exert immunomodulatory effects via interaction with several receptors such as CXCR4 and induction of signaling pathways (NFkB, MAPK) in immune cells (18). A further anti-infectious property of Vit-D is a result of the activation of the phagocyte NADPH oxidase (NOX), which induces an increase in reactive oxygen species (ROS) production (19, 20).

Vit-D is known to shift the T-cell response from a T helper 1 (Th1) to a Th2-mediated cell response, and thereby reduce inflammation and promote an immunosuppressive state (21–23). Moreover, it promotes *in vitro* the regulatory T cells (Treg) differentiation via an indoleamine 2,3-dioxygenase (IDO)-dependent pathway (24, 25). Thus Vit-D may be an important immune response regulator, notably in vaccine and infection challenges (26, 27).

The public health strategy for influenza is to reduce severe outcomes such as hospitalization and death by recommending annual vaccinations, particularly for people over 65 years old (28, 29). However, the vaccine efficacy is lower for older persons (17–53%) than for young adults (70–90%) (30, 31). This could be related to the Vit-D deficiency as reported in previous clinical studies (32–34). To our knowledge, no Vit-D supplementation trial has yet been conducted in Vit-D-deficient elderly populations with the aim of improving vaccination efficacy.

Considering these data, we assessed the impact of Vit-D supplementation on the immune response to influenza vaccination in Vit-D-deficient elderly volunteers by evaluating (i) cathelicidin status, and (ii) antibody response to vaccine, cytokine production, IDO activity, lymphocyte polarization and ROS production.

## Materials and Methods

### Volunteer Recruitment and Randomization

Eligible volunteers were over 65 years old and accepted Vit-D or placebo supplementation and influenza vaccination. Exclusion criteria included prior hypersensitivity to Vit-D (in the previous year), ongoing Vit-D supplementation, previous side effects, and complications after vaccination, hypercalcemia (>2.6 mmol/L), dysparathyroidism, renal impairment, and long-term treatment with bisphosphonates, corticosteroids, or fibrates.

Volunteers were randomly assigned to blocks of four by sex and age using a computerized random-sequence-generation program run by an independent researcher who was not involved in the data collection, analysis, or reporting. For the supplementation, placebo and Vit-D doses were identical in appearance to maintain blinding, and all participants, investigators, and outcome assessors remained blinded until after all of the data was inputted.

### Protocol Design

This randomized double-blind controlled trial was authorized by the ethics committee (Comité de Protection des Personnes Sud-Est 6, Clermont-Ferrand, France) and the French state authority (Agence Nationale de Sécurité du Médicament). It was registered on EudraCT under ref. 2012-005658-52 and on clinicaltrials.gov as NCT01893385. At the inclusion visit the volunteers gave fully informed written consent, and then blood samples were taken to determine serum Vit-D levels and the biological parameters required to validate eligibility criteria: blood cell count, and usual plasma and urinary levels of calcium, phosphorus, creatinine, liver enzymes (AST, ALT), glucose, and total proteins. Based on serum Vit-D data, the volunteers were grouped as follows: (i) persons with a serum Vit-D level greater than or equal to 30 ng/mL: these individuals were excluded, and advised to accept an influenza vaccine in autumn; (ii) persons with a Vit-D level below 30 ng/mL: these individuals were randomly assigned to one of two groups: (1) a supplemented group (D) receiving six Vit-D doses (Uvedose® 100,000 IU, 1 vial/15 days, Crinex Lab.) over 3 months, followed by an influenza vaccination; (2) a control group (P) receiving a placebo (1 vial/15 days, Crinex Lab.) over 3 months, followed by an influenza vaccination. The participants' compliance was verified by restitution of all empty vials at each visit.

Influenza vaccination was carried out using the IM vaccine Vaxigrip® (Sanofi Pasteur), which provides seroprotection against all seasonal influenza strains, namely A/California/7/2009 (H1N1, pdm09), A/Texas/50/2012 (H3N2), and B/Massachusetts/2/2012 (Yamagata lineage). The volunteers committed not to change their eating habits, and were assessed at three different stages: at inclusion (V1), after 3 months of supplementation (V2), and 1 month after vaccination (V3). A survey of side effects and complications was performed at each visit and by telephone interview. For the D group, serum Vit-D concentration, calcemia, and calciuria were monitored after 2 months of supplementation. The biological parameters discussed in the following paragraphs were measured at each visit (V1, V2, and V3).

### Serum Vit-D and Cathelicidin Assays

Serum 25-(OH)D was measured by chemiluminescence immunoassay (Liaison XL analyzer, DiaSorin). Serum cathelicidin (LL-37 protein) was quantified using a double-sandwich ELISA, following the manufacturer's guidelines (Hycult Biotechnology—HK321).

### Serum Antibody Quantification

Vaccine response was assessed at two points (V2 and V3) by measuring hemagglutination inhibition (HAI) antibody (Ab) titers against the influenza vaccine antigens. The HAI test was performed in microplates by incubating serum with the 2013-2014 influenza reference strains (H1N1, H3N2, and Yamagata), following the WHO procedure. Inter assay quality control was performed with reference antisera as positive controls. Erythrocyte controls allowed adjustments in incubation time and were performed on each plate. Each field isolate antigen and the control antigens have been tested with a negative serum control. The HAI Ab titer was defined as the highest dilution of serum inhibiting the agglutination of guinea pig erythrocytes (Charles River Lab.). In accordance with the European Agency for the Evaluation of Medicinal Products' guidelines (35), data was expressed in 3 ways: geometric mean titer (GMT) with a 95% confidence interval; seroconversion rate (percentage of subjects achieving at least a 4-fold increase, or an increase from >10 to 40 in HAI Ab titer for seronegative subjects); and seroprotection rate, i.e., percentage of subjects reaching an HAI Ab titer 40.

### Plasma Cytokine Assays

The concentrations of plasma cytokines were quantified using a multiplex assay (Milliplex, Millipore), following the manufacturer's instructions: IL-5, IL-6, IL-10, IL-13, IL-17A, IFNγ, TNFα (Hcytomag-60K-7plex). For IL-23 and TGFβ, a singleplex assay was used (Tgfbmag-64K-01-1plex).

### Serum Tryptophan (Trp) and Kynurenine (Kyn) Assays

IDO activity was determined for half of the volunteers (P group, *n* = 10; D group, *n* = 9), and estimated by the ratio of Kyn to Trp serum concentrations as described previously (36). Shortly after deproteinization serum samples were analyzed using HPLC on a reverse phase C18 column (Thermo Scientific). Kyn and Trp concentrations (μmol/L) were calculated using the area under the curve method.

### Lymphocyte Phenotyping and ROS Production

Fresh leukocytes were obtained from volunteers' blood samples. After hemolysis, leukocytes were separated on a discontinuous Ficoll–Hypaque density gradient (Histopaque® 1077 and 1119; Sigma) as described previously (37). Lymphocyte population was tested for purity (>95%) and viability (>95%) and phenotyped by flow cytometry (LSRII, BD Biosciences) using antibody panels: anti CD3-VioBlue (T-cell), anti CD4-APC (Th), anti CD25-APC (activated T-cell), anti CD183 (CXCR3)-PE-Vio770 (Th1), anti CD294 (CRTh2)-PE (Th2), anti CD196 (CCR6) PercP-Cy5.5 (Th17, Biolegend, San Diego), and anti CD127-FITC (Treg, Myltenyi BioTec, Paris). FACS gating strategy was illustrated in Figure 7A: compensations and controls used the FMO (Fluorescence Minus One) procedure with corresponding antibody isotypes.

ROS production of polymorphonuclear cells (PMN) was quantified from hemolyzed blood by an intracellular fluorescent probe [2′,7′-dichlorofluorescein (DCF) 1 μM, Sigma-Aldrich] using flow cytometry, as described previously (3).

### Sample Size and Study Power

The primary outcome of the trial was the difference in serum cathelicidin levels between the placebo and Vit-D study arms after 3 months of supplementation. To detect this significant difference, the calculation was based on the hypothesis that the mean ± SD baseline serum cathelicidin concentration was 13.3 ± 1.8 ng/ml and that the Vit-D supplementation would cause a difference of 1.26 ± 2.1 ng/ml in cathelicidin between the 2 arms (80% power with α = 0.05). Taking these assumptions into account, we calculated a group size of 42 participants per arm.

### Statistical Analysis

Data are expressed as mean ± SEM. Statistical analysis was performed using GraphPad Prism® 5.03 for Windows (GraphPad Software Inc., San Diego, CA, USA). Vit-D supplementation and period effects were analyzed by two-way ANOVA followed by a Bonferroni *post hoc* test. Differences within groups were determined by a paired Student *t*-test or a Wilcoxon matched-pairs signed-ranks test. Differences between groups were tested by an independent Student *t*-test or a Mann-Whitney *U*-test. Differences were considered statistically significant at *p* < 0.05. The relationship between serum Vit-D and cathelicidin data was assessed using a Pearson correlation (significant threshold: *p* < 0.05).

## Results

### Volunteer Inclusion and Follow-Up

The volunteers were recruited over 2 years (2013 and 2014) on a similar schedule: the first visit (V1) in June, the second in October at the end of supplementation and for vaccination (V2), and the third in November, 28 days after vaccination (V3). Because of recruitment difficulties, the number of volunteers selected (*n* = 47) was half the original intended sample size (*n* = 84). An intermediary analysis was conducted, and showed a discrepancy between the initial hypothesis and the results obtained for cathelicidin. For these reasons, and because the vaccine strains were set to change the following year (2015), we decided to end recruitment.

**Figure 1:**
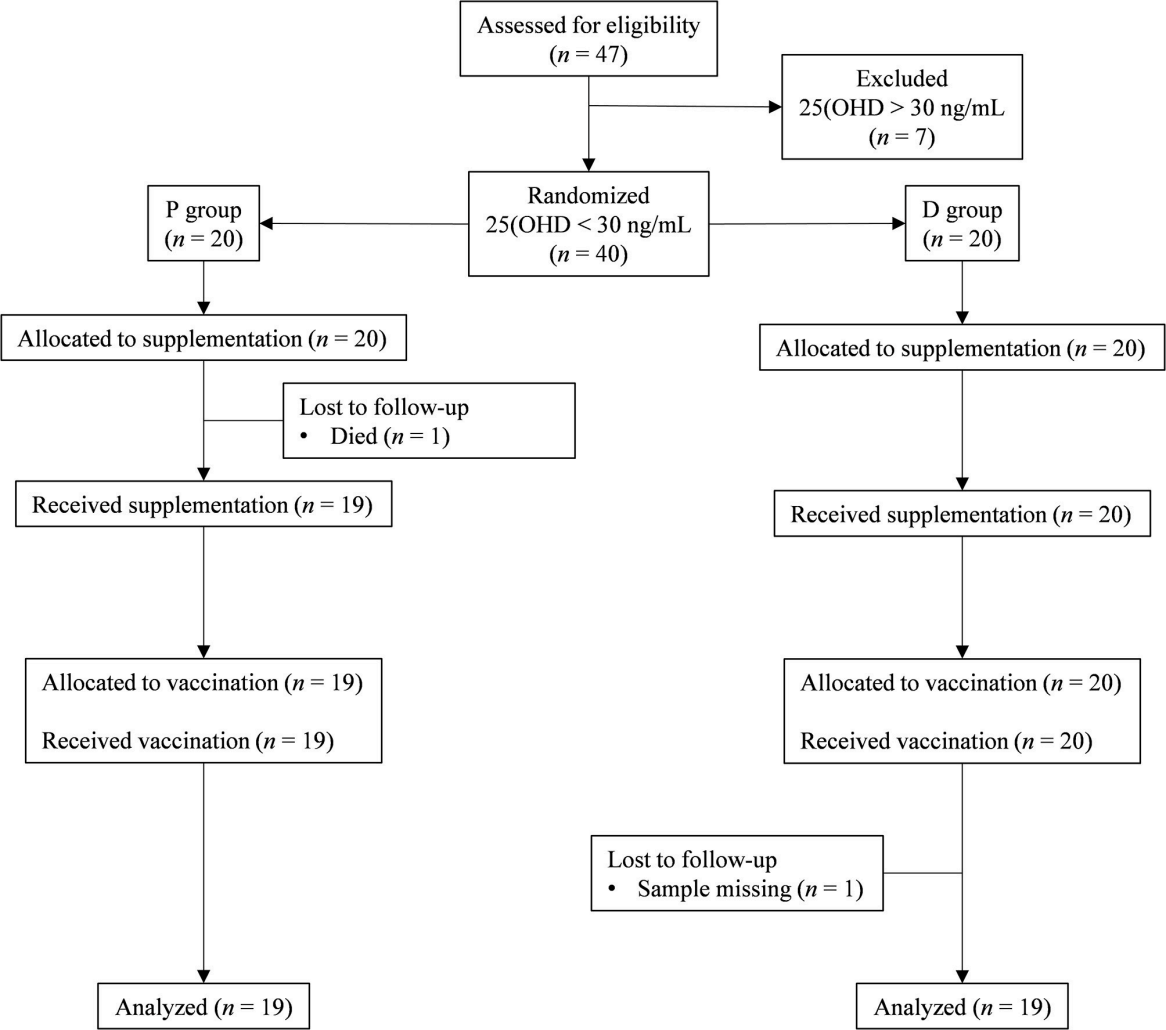
CONSORT flow chart. The flow of participants through the trial is represented by a diagram, as suggested by the CONSORT group. P group, Placebo supplementation; D group, Vit-D supplementation.

Of the 47 eligible volunteers, 38 Vit-D-deficient individuals were analyzed in the placebo (*n* = 19) and Vit-D (*n* = 19) groups; the causes of drop-out are indicated in the flow chart (Figure 1). Volunteer characteristics at inclusion showed no difference between the two groups (Table 1). All plasma biochemical markers were within the normal ranges.

**Table 1:** Characteristics of healthy volunteers at inclusion^1^.

	**P group (*n* = 19)**	**D group (*n* = 19)**	***p*^2^**
**ANTHROPOMETRIC PARAMETERS**			
Sex ratio, *m/f*	12/8	11/9	0.99
Age, *y*	70 ± 6	72 ± 5	0.99
Height, *cm*	166 ± 7	165 ± 8	0.99
Weight, *kg*	75 ± 12	72 ± 14	0.99
Body mass index, *kg/m^2^*	27.3 ± 3.9	26.3 ± 3.5	0.99
Abdominal perimeter, *cm*	99 ± 10	96 ± 12	0.99
**BIOLOGICAL PARAMETERS**			
Sodium, *mmol.L^−1^*	140 ± 2	140 ± 2	0.99
Potassium, *mmol.L^−1^*	4 ± 0.4	4 ± 0.2	0.99
Chloride, *mmol.L^−1^*	106 ± 2	104 ± 2	0.99
Total proteins, *g.L^−1^*	76 ± 3	75 ± 4	0.99
Glucose, *mmol.L^−1^*	5.2 ± 0.9	5.0 ± 0.9	0.99
Calcium, *mmol.L^−1^*	2.2 ± 0.1	2.2 ± 0.1	0.99
Urea, *mmol.L^−1^*	6.3 ± 1.5	6.0 ± 1.1	0.99
Creatinine, *μmol.L^−1^*	72 ± 13	76 ± 14	0.99
Phosphorus, *mmol.L^−1^*	0.9 ± 0.1	0.9 ± 0.1	0.99
AST, *UI.L^−1^*	22 ± 7	22 ± 7	0.99
ALT, *UI.L^−1^*	27 ± 9	27 ± 9	0.99
25-OH vitamin D, *ng.mL^−1^*	19.7 ± 5.9	20.7 ± 5.7	0.99

*P group, Placebo supplemented group; D group, Vit-D supplemented group*.

1 *Data are expressed as mean ± SD*.

2 *p-values were determined using a Mann-Whitney U-test*.

A telephone follow-up after 6 weeks confirmed subjects' clinical safety, and the absence of side effects from the supplementation. Volunteer compliance for the Vit-D or placebo supplementation, assessed by serum vitamin D quantification, was satisfactory. A biological test after 2 months of supplementation showed neither serum Vit-D >75 ng/mL nor hypercalcemia nor hypercalciuria.

### Serum Vitamin D

**Figure 2:**
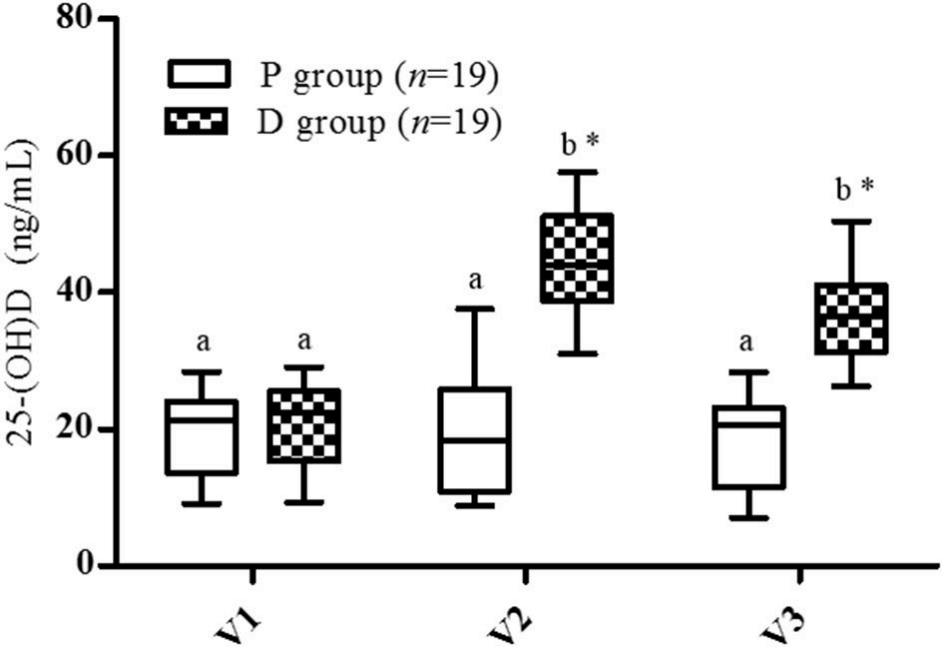
Vitamin D serum levels. V1, inclusion; V2, end of supplementation and vaccination; V3, 28 days post-vaccination. Data are expressed as mean ± SD. Statistical analysis was performed by two-way ANOVA for the supplementation and time effect followed by Bonferroni *post hoc* test (*p* < 0.05); ^*^Significantly different between groups at the same visit (*p* < 0.05); ^a,b^ Significantly different between visits in the same group (*p* < 0.05).

At inclusion, no statistical difference in serum Vit-D level was observed between the groups (P: 19.7 ± 5.9 ng/mL, D: 20.7 ± 5.7 ng/mL, Figure 2). For the P group, no variation was found during the entire protocol (V1: 19.4 ± 6.24 ng/mL, V2: 19.1± 7.9 ng/mL, V3: 18.1 ± 6.7 ng/mL). For the D group, a significant increase was observed after the supplementation period (V1: 20.7 ± 5.7 to V2: 44.3 ± 8.6 ng/mL, *p* < 0.001), and for all subjects, 25-(OH)D concentration was >30 ng/mL at V2. The highest serum 25-(OH)D level was 58 ng/mL with the 600,000 IU cumulative Vit-D dose. This supplementation was demonstrated to be safe: no variation in plasma or urinary calcium levels and no clinically relevant adverse effects were observed. One month after the end of the supplementation (V3), no significant decrease in serum Vit-D level was observed in the D group (V3: 36.5 ± 6.3 vs. V2: 44.3 ± 8.6 ng/mL).

### Serum Cathelicidin

**Figure 3:**
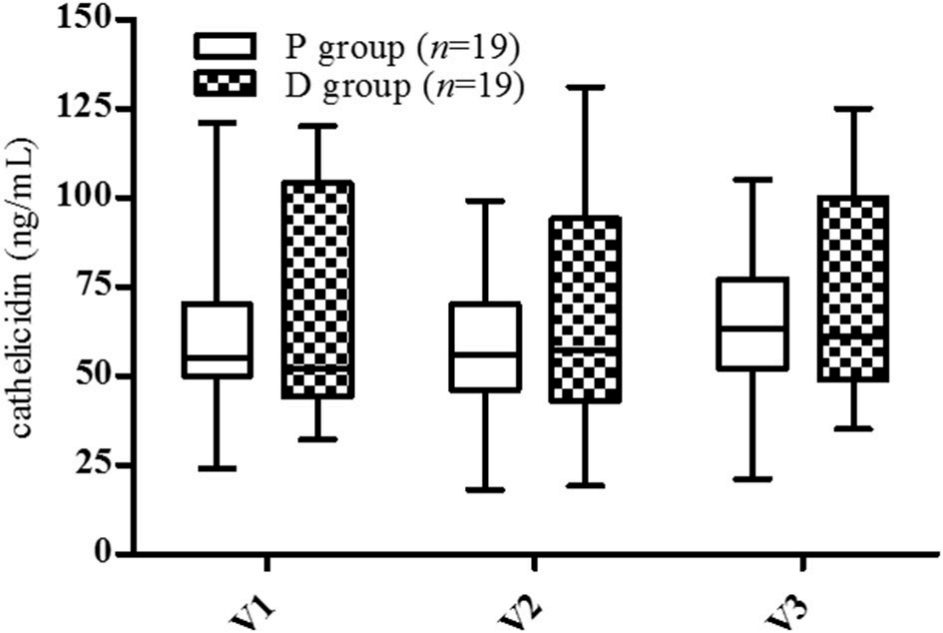
Cathelicidin serum levels. V1, inclusion; V2, end of supplementation and vaccination; V3, 28 days post-vaccination. Data are expressed as mean ± SD. No statistical difference was observed by two-way ANOVA (*p* < 0.05).

At inclusion (V1), no statistical difference between groups was observed in serum cathelicidin levels (P: 62.0 ± 5.5 ng/mL, D: 66.2 ± 6.9 ng/mL, Figure 3). The highest and lowest values were similar (P: 24-121 ng/mL, D: 25-120 ng/mL), although there was a greater dispersion of values in the P group than in the D group. No variation was observed in any period (V2, V3) in either group.

**Figure 4:**
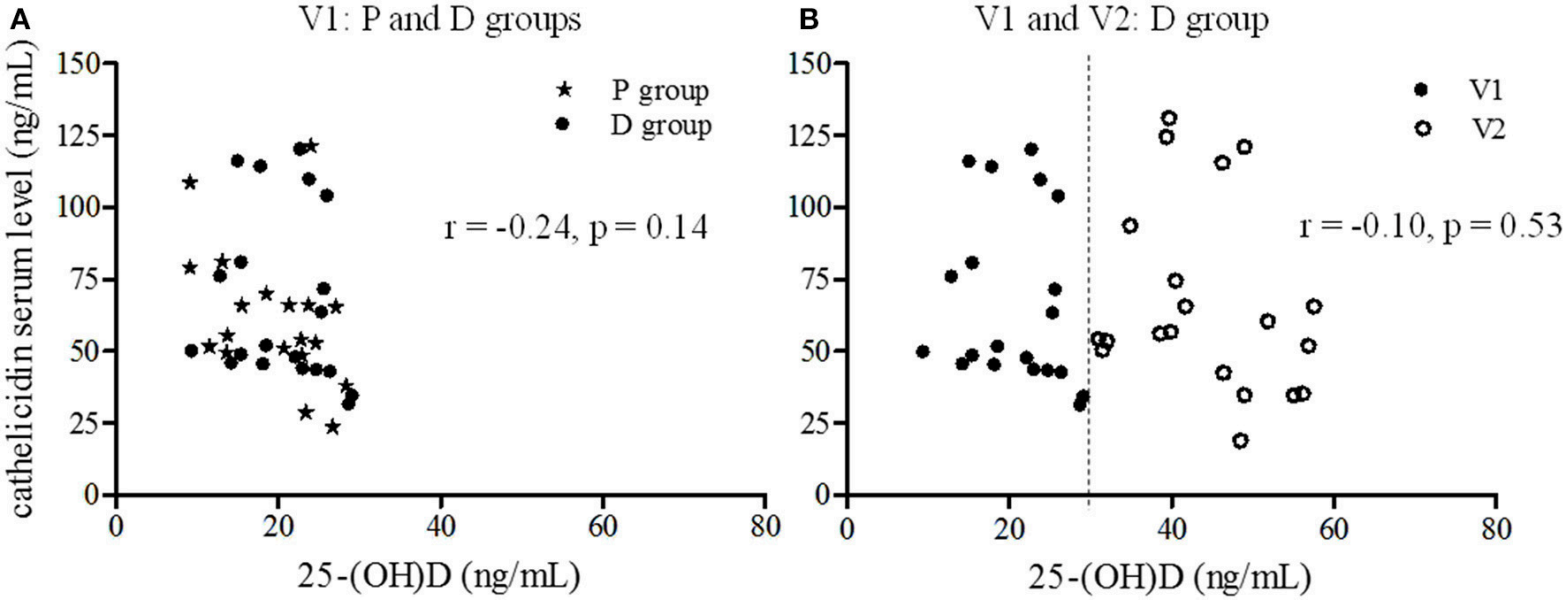
Correlation between 25-(OH)D and cathelicidin serum levels. The relationship between cathelicidin and 25-(OH)D serum levels was estimated using Pearson correlation. **(A)** Correlation at inclusion (V1) for all volunteers (star P group, circle D group) (*r* = −0.24, *p* = 0.14). **(B)** Correlation before (V1: dark circle) and after the supplementation period (V2: light circle) for D group (*r* = −0.10, *p* = 0.53).

No correlation was found between serum Vit-D and cathelicidin levels for volunteers at inclusion (*r* = **–**0.24, *p* = 0.14, Figure 4A). Moreover, considering the data before and after supplementation for the D group, no significant relationship between cathelicidin and 25-(OH)D serum levels was observed (*r* = **–**0.10, *p* = 0.53, Figure 4B).

### Antibody Response to Influenza Vaccination

**Figure 5:**
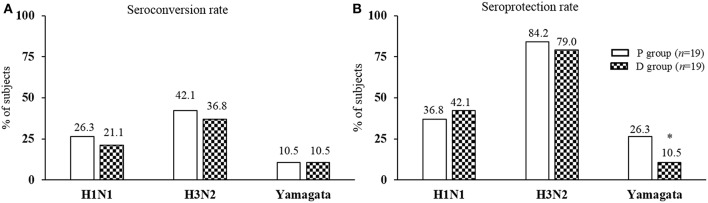
Post-vaccination seroconversion and seroprotection rates. **(A)** Seroconversion rate: percentage of subjects achieving at least a 4-fold increase or an increase from >10 to 40 in Ab titer for seronegative subjects; **(B)** Seroprotection rate: percentage of subjects reaching an Ab titer 40. ^*^Significantly different between P and D group using Mann-Whitney *U*-test (*p* < 0.05).

Ab titers to inactivated influenza virus strains are presented in Table 2. The Ab titers increased significantly for the three strains after vaccination in both P and D groups except for H1N1 in D group, because of data dispersion (Table 2; *p*^2^, *p*^3^). For the pre-vaccination Ab titers, there was no significant difference between the groups for any strain (Table 2; *p*^4^). Nor was there any significant difference for post-vaccination Ab titers, except for the H3N2 strain which was significantly lower in the D than the P group (Table 2; *p*^5^). No significant differences were observed after vaccination between P and D groups in either seroconversion (Figure 5A) or seroprotection (Figure 5B) rates, except for Yamagata seroprotection (P: 26.3% vs. D: 10.5%, *p* < 0.05). This data needed to be stratified with regards to volunteers' serologic status before vaccination.

**Table 2:** Antibody response to inactivated influenza virus vaccine in all volunteers^1^.

	**Ab titers—P group (*****n*** **=** **19)**				**Ab titers—D group (*****n*** **=** **19)**						
**Vaccine strain**	**Pre-vaccination**	**Post-vaccination**	***p^2^***	**Ratio post/pre**	**Pre-vaccination**	**Post-vaccination**	***p^3^***	**Ratio post/pre**	***p^4^***	***p^5^***	***p^6^***
H1N1	9.0 (3.2–14.8)	20.7 (12.7–28.7)	0.003	2.3 ± 1.3	12.4 (4.6–20.2)	20.0 (11.0–29.0)	0.066	1.6 ± 0.6	0.310	0.905	0.291
H3N2	29.4 (12.9–45.9)	107 (86.5–127)	0.0005	3.5 ± 2.6	17.9 (6.0–29.8)	51.6 (36.5–66.7)	0.001	3.0 ± 1.2	0.225	0.046	0.397
Yamagata	8.0 (6.2–9.8)	12.4 (6.2–18.6)	0.022	1.5 ± 0.6	6.2 (5.8–6.6)	10.0 (3.8–16.2)	0.021	1.6 ± 0.8	0.345	0.651	0.714

*P group, Placebo supplemented group; D group, Vit-D supplemented group*.

1 *Ab titers are expressed as GMT (95% CI)*.

2,3 *Determined using a paired Wilcoxon test for intra-group differences between pre- and post-vaccination Ab titers in the P group (p^2^) and in the D group (p^3^)*.

4,5,6 *Determined using Mann-Whitney U-test for inter-group differences in pre-vaccination Ab titers (p^4^), in post-vaccination Ab titers (p^5^) and Ab titers ratio (p^6^) between P and D groups*.

**Figure 6:**
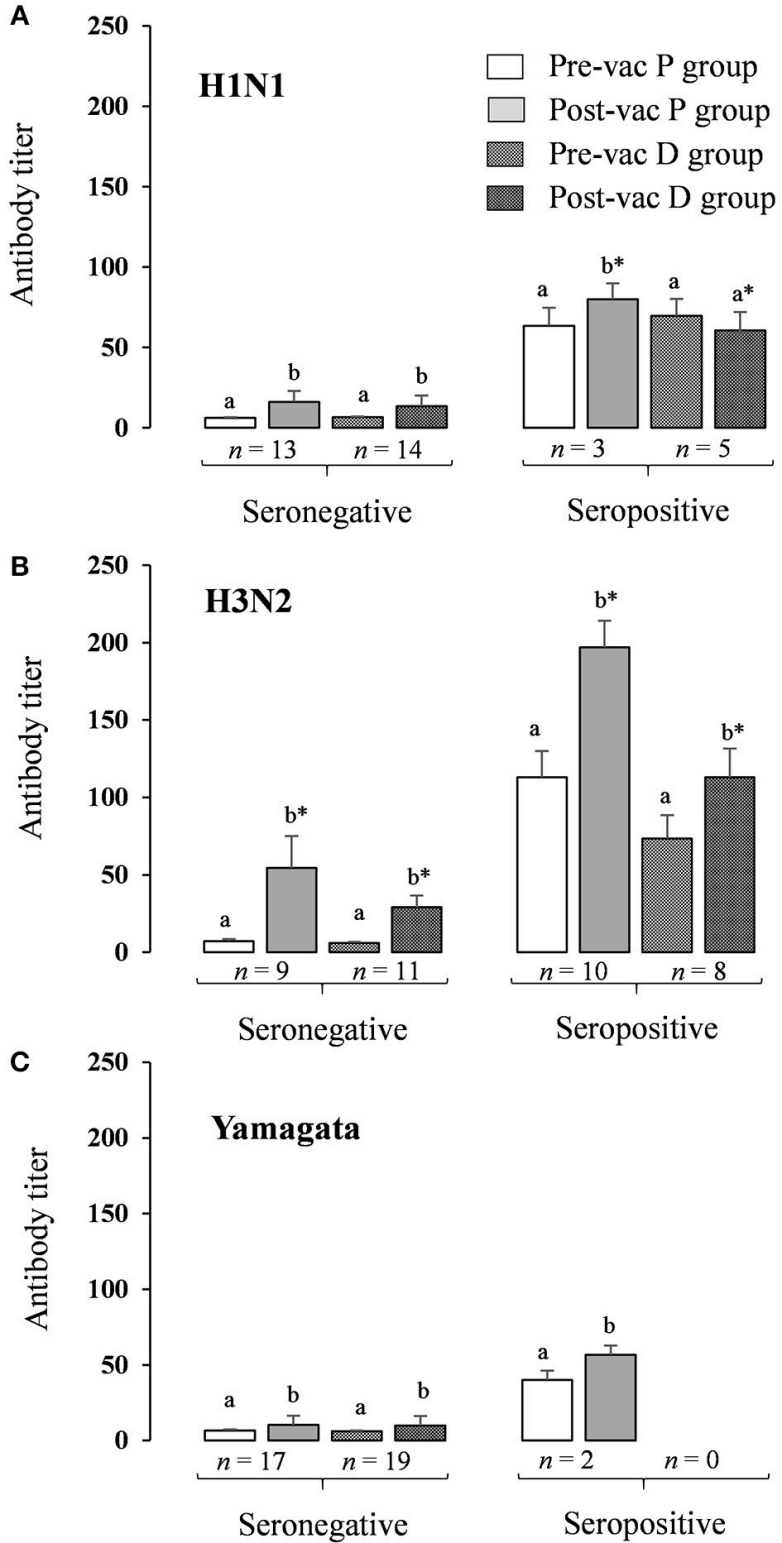
HAI antibody titer response to inactivated influenza virus vaccine in seronegative and seropositive volunteers. HAI antibody titers stratified by pre- and post-vaccination status. **(A)** Antibody titers for H1N1 pandemic influenza A (A/California/7/2009, H1N1pdm09); **(B)** Antibody titers for H3N2 pandemic influenza seasonal influenza A (A/Texas/50/2012, H3N2); **(C)** Antibody titers for Yamagata seasonal influenza B (B/Massachusetts/2/2012, Yamagata lineage). Results are expressed as GMT (95% IC); ^a,b^Significantly different between pre- and post-vaccination titers in the same group using paired Wilcoxon test (*p* < 0.05); ^*^Significantly different for a same period (pre- or post-vaccination) between P group and D group using Mann-Whitney U-test (*p* < 0.05).

For the seronegative volunteers, the Ab titer for each strain increased significantly (*p* < 0.05) after vaccination in both P and D groups, with a significantly lower level of H3N2 in the D than in the P group (*p* < 0.05; Figures 6A–C). For seropositive volunteers, the Ab titer of the three strains increased significantly (*p* < 0.05) after vaccination in the P group, although in the D group only the H3N2 strain increased significantly (Figures 6A–C). Looking at the post-vaccination data as a whole, the seronegative volunteers had fewer Ab titers than the seropositive volunteers.

### Cytokine Profile

The plasma cytokine levels were determined to evaluate the T-cell response at each period (V1, V2, and V3): Th1 (IFNγ, TNFα), Th2 (IL-5, IL-6, IL-10, IL-13), Th17 (IL-17A, IL-23), and Treg (TGFβ) (Table 3). No data is available for IL-10 and IL-13 because sample concentrations were below the limit of quantification.

**Table 3:** Cytokine plasma levels^1^.

	**P group (*****n*** **=** **19)**			**D group (*****n*** **=** **19)**			***p***^2^		
	**V1**	**V2**	**V3**	**V1**	**V2**	**V3**	**Vit-D**	**Visit**	**Interaction**
IFNγ (pg/mL)	3.76 ± 0.99	2.48 ± 0.74	3.16 ± 1.09	3.59 ± 1.13	2.99 ± 1.06	3.35 ± 1.15	0.067	0.384	0.685
TNFα (pg/mL)	3.89 ± 0.58	3.87 ± 0.95^3^	3.87 ± 0.88	3.05 ± 0.44	2.49 ± 0.44^3^	2.81 ± 0.57	0.040	0.905	0.919
Il-5 (pg/mL)	1.00 ± 0.53	0.87 ± 0.37	0.72 ± 0.18	0.88 ± 0.29	0.66 ± 0.14	0.91 ± 0.22	0.868	0.847	0.800
Il-6 (pg/mL)	1.49 ± 0.32	1.11 ± 0.18	1.13 ± 0.20	1.09 ± 0.16	0.90 ± 0.01	0.86 ± 0.03	0.046	0.179	0.866
IL-17A (pg/mL)	0.71 ± 0.23	0.57 ± 0.04	0.90 ± 0.29	1.07 ± 0.33	0.81 ± 0.15	1.03 ± 0.19	0.511	0.384	0.474
IL-23 (pg/mL)	303 ± 101	250 ± 114	295 ± 194	337 ± 103	322 ± 99.8	236 ± 95.0	0.870	0.902	0.856
TGFβ (ng/mL)	9.88 ± 2.23	14.2 ± 3.87	11.5 ± 3.70^4^	13.6 ± 2.55	11.4 ± 2.27	20.8 ± 3.37^4^^,^^5^	0.175	0.321	0.145

*P group, Placebo supplemented group; D group, Vit-D supplemented group; V1, inclusion; V2, end of supplementation and vaccination; V3, 28 days post-vaccination*.

1 *Results are expressed as mean ± SEM*.

2 *Statistical analysis was performed using a two-way ANOVA to discriminate between the supplementation effect (Vit-D) and the period-related effect (Visit) (p < 0.05). When the ANOVA indicated significant interactions, the Bonferroni post hoc test was used*.

3 *Significant difference between P and D groups at V2 using Mann-Whitney U-test (p < 0.05)*.

4 *Significant difference between P and D groups at V3 using Mann-Whitney U-test (p < 0.05)*.

5 *Significant difference for D group between V2 and V3 using paired Wilcoxon test (p < 0.05)*.

The IFNγ and IL-5 plasma levels were similar irrespective of group or period, with no variation in the IFNγ /IL-5 ratio (P-V1: 7.16 ± 2.11, V2: 4.81 ± 1.60; D-V1: 7.48 ± 2.33, V2: 5.50 ± 2.11). Significantly lower levels of TNFα (V2, *p* = 0.0478, *U*-test) and IL-6 (*p* = 0.046, ANOVA) were observed in the D than in the P group, with no significant variation in TNFα/IL-6 ratio (P-V1: 3.06 ± 0.45, V2: 4.21 ± 1.14; D-V1: 3.22 ± 0.53, V2: 2.74 ± 0.45).

No change was observed in the Th17-cytokine response (IL-17A and IL-23) either between groups or over different periods. The level of TFGβ was significantly higher in the D than in the P group after vaccination (V3, *p* = 0.0028, *U*-test) and in V2 than V3 in the D group (*p* = 0.0084, Wilcoxon test).

### Indoleamine-2,3-deoxygenase (IDO) Activity

We evaluated serum IDO activity through the Kyn to Trp concentrations ratio. No significant differences were observed in IDO activity in any group or period (Table 4).

**Table 4:** Indoleamine-2,3-deoxygenase serum activity^1^.

	**P group (*****n*** **=** **10)**			**D group (*****n*** **=** **9)**			***p***^2^		
	**V1**	**V2**	**V3**	**V1**	**V2**	**V3**	**Vit-D**	**Visit**	**Interaction**
Kyn (μmol/L)	2.4 ± 0.2	2.1 ± 0.6	2.6 ± 0.4	2.4 ± 0.1	2.0 ± 0.2	2.2 ± 0.2	0.192	0.363	0.882
Trp (μmol/L)	47.0 ± 2.5	42.1 ± 2.3	46.9 ± 3.5	46.9 ± 2.8	43.8 ± 2.8	44.5 ± 3.8	0.309	0.918	0.811
Kyn/Trp ratio (x100)	5.2 ± 0.5	5.0 ± 0.3	5.3 ± 0.3	5.1 ± 0.6	4.5 ± 0.5	5.2 ± 0.8	0.533	0.564	0.888

*P group, Placebo supplemented group; D group, Vit-D supplemented group; V1, inclusion; V2, end of supplementation and vaccination; V3: 28 days post-vaccination*.

1 *IDO activity was estimated by Kyn/Trp ratio. Data are expressed as mean ± SEM*.

2 *Statistical analysis was performed using a two-way ANOVA to discriminate between the supplementation effect (Vit-D) and the period-related effect (Visit) (p < 0.05)*.

### T Cell Phenotypes and PMN ROS Production

**Figure 7:**
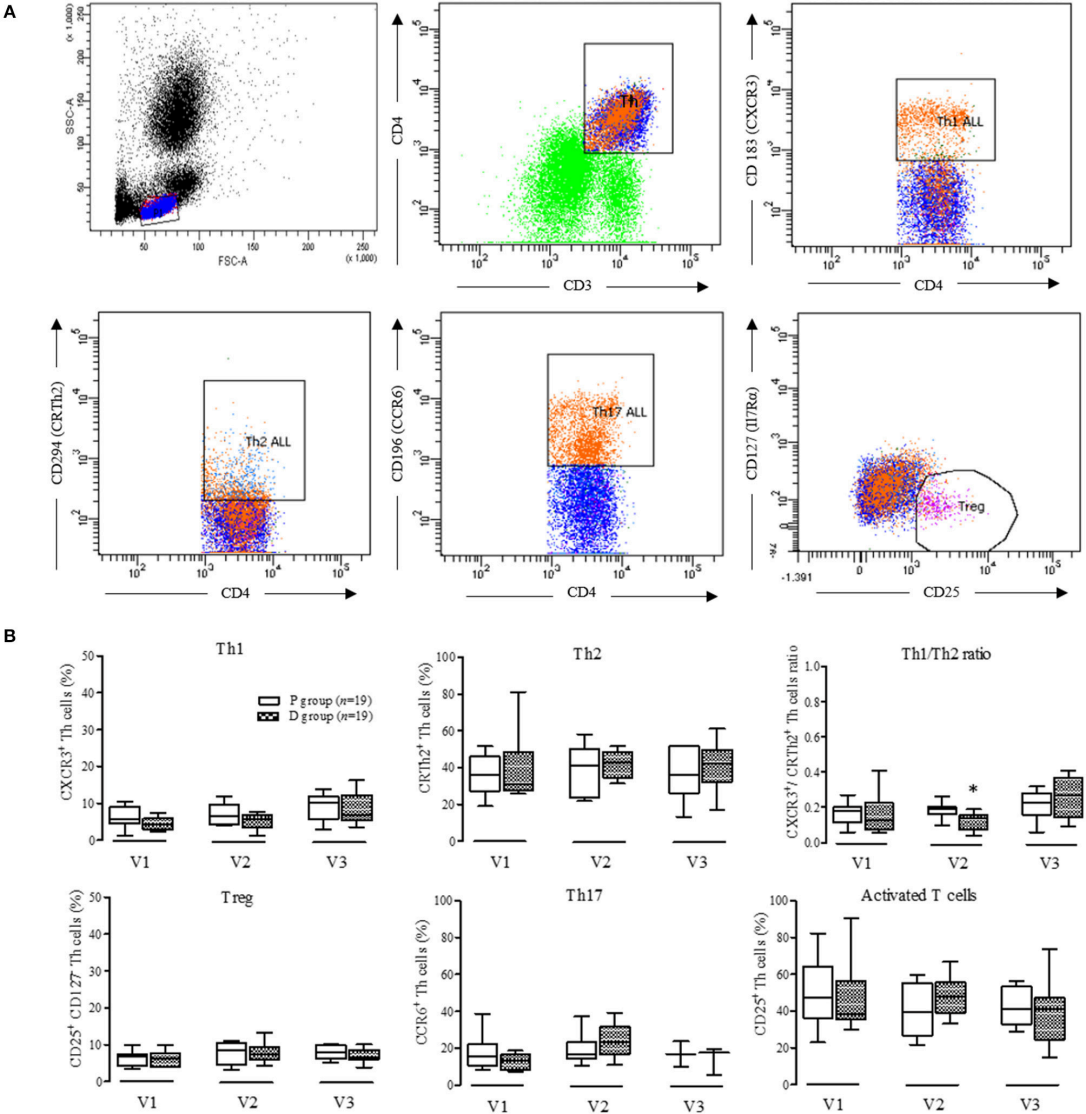
Phenotyping of peripheral blood CD3^+^CD4^+^ T cells. **(A)** Gating strategy of a representative sample used to identify T helper cells (Th. CD3^+^CD4^+^), Th1 cells (CD4^+^, CD 183^+^), Th2 cells (CD4^+^,CD294^+^), Th17 cells (CD4^+^, CD196+), and Treg cells (CD25^+^, CD127^−^); **(B)** Proportion from the different T helper and Treg cells in D group compared to controls. V1, inclusion; V2, end of supplementation and vaccination; V3, 28 days post-vaccination; Data are expressed as mean ± SD. No statistical difference was observed by two-way ANOVA (*p* < 0.05); ^*^Determined using Mann-Whitney *U*-test between P and D groups (*p* < 0.05).

Lymphocyte polarization phenotyping was based on co-expression of CD3, CD4, and specific surface markers of Th1 (CXCR3), Th2 (CrTh2), Th17 (CCR6), and Treg (CD125^+^, CD127^−^) (Figure 7B). Percentages of Th cells did not vary over periods or between groups, except for a significant decrease in the Th1 to Th2 ratio observed when comparing the D group to the P group at the end of Vit-D supplementation (V2).

The PMN basal ROS production, expressed in fluorescence arbitrary unit, did not vary over periods or between groups (P - V1:29.0 ± 2.8; V2: 29.1± 2.9; V3: 36.8 ± 3.8; D - V1: 28.5 ± 2.4; V2: 35.6 ± 3.6; V3: 41.3 ± 4.6).

## Discussion

By analyzing several immune biomarkers, this trial assessed the effects of Vit-D supplementation on the response to influenza vaccination in Vit-D-deficient elderly persons.

The study carries limitations that warrant consideration. Firstly, the differences in volunteers' vaccine status before vaccination may have limited the ability to observe the effects of Vit-D supplementation on influenza vaccination response. Secondly, the small sample size, which could mean the study was underpowered to detect changes in serum cathelicidin levels despite volunteers' well-defined Vit-D deficiency status and the significant (2-fold) increase in Vit-D level in the supplemented group. The lack of a reference analytical method and physiological ranges for serum cathelicidin may further compound this issue. Consequently, the study power calculated on cathelicidin variations with Vit-D supplementation was reduced from 80 to 47%.

At the end of Vit-D supplementation (100,000 UI × 6), the mean change in serum Vit-D level ranged from +16.7 to +37.1 ng/mL, corresponding to 0.23–0.52 ng/mL for 100 IU. This is in line with data from Schleck et al. (38), who reported an increase of 0.30–0.46 ng/mL for 100 IU after a 12-week treatment. The serum Vit-D level quickly decreased after supplementation ended (by approximately 18% in 4 weeks), suggesting a short-lived efficacy. In our conditions, the Vit-D supplementation induced no adverse events, and others have demonstrated that doses larger than those used here are safe (39).

The antimicrobial properties of Vit-D have been extensively studied with respect to tuberculosis, where Vit-D enhances cathelicidin production and autophagy (40). These effects have also been described in viral infections such as HIV and respiratory diseases (40). At inclusion in our trial, volunteers showed a wide range of cathelicidinemia (from 29 to 121 ng/mL).In healthy elderly Chinese persons, Yang et al. reported, using the same ELISA method, lower levels of cathelicidin (20.7 ± 5.8 ng/mL) associated with Vit-D concentrations (18.1 ± 9.4 ng/mL) similar to those observed in our study (41). Using another ELISA method in a healthy population, Bhan et al. (42), and Dixon et al. (43) established a positive correlation between serum cathelicidin and Vit-D levels when 25-(OH)D concentration was lower than 32 ng/mL. However, we did not find this correlation, despite subjects' 25-(OH)D concentrations being under 32 ng/ml. Similarly, several authors reported no change in circulating cathelicidin (44, 45), although Vit-D supplementation resulted in leukocyte increased cathelicidin mRNA expression (46). There could be a Vit-D-independent regulation of cathelicidin expression, or of cleaving activity of serine proteinase 3, or of cathelicidin proteolysis (12). Also, owing to its polycationic structure, blood free cathelicidin is rapidly bound to negatively-charged compounds, and so is unavailable for quantification. These various factors may explain why it is so difficult to demonstrate a Vit-D-induced increase in serum cathelicidin concentration.

The effect of 25-(OH)D supplementation on the humoral immune response to influenza vaccination was evaluated. It showed no effect on Ab production in either seroprotection or seroconversion. This finding is consistent with two randomized controlled trials of Vit-D supplementation in influenza-vaccinated healthy adults (34) and adolescents (47). These trials did not characterize the Vit-D status prior to the supplementation, unlike our study.

The volunteers' seroprotection (11–84%) and seroconversion rates (10–42%) were in line with data from the CDC, suggesting a clinical efficacy of 17–53% in elderly persons (30). The pre-vaccination Ab status must be taken into account when considering the effect of Vit-D supplementation on vaccine response. Since almost all of the volunteers had been vaccinated in the previous year, high levels of pre-vaccination Ab titer were expected. The pre-vaccination Ab titers for type A strains in both P and D groups were lower than those in previous reports (48). Hirota et al. (48) showed a significant inverse association of pre-vaccination serologic status with both titer fold rise and response rate in the serum Ab. In our study, a subgroup analysis of pre-vaccination Ab titers showed that the seroconversion rate was not affected by Vit-D-supplementation, but was lower for seronegative subjects than for seropositive ones. McElhaney reported that elderly persons who had been vaccinated every year were better protected than those who were vaccinated for the first time, suggesting that the absolute post-vaccination Ab titer is a better marker of protection than the Ab mean fold increase (49). In our study, the “intact” Ab response to the vaccine, defined by Hara et al. (50) as one showing post-vaccination HAI titer ≥ 40 for at least one strain, was similar in both groups (84%).

Considering the innate immune response, Vit-D supplementation induced a shift to a Th2- cytokine response as previously described (increased levels of IL-4, IL-5, IL-10, and reduced levels of IL-2, IFNγ, and TNFα) (21, 22). In our study, we confirmed that Vit-D supplementation significantly reduced the plasma level of TNFα and IL-6. Limited data from observational studies lends support to an anti-inflammatory role of vitamin D. In an observational study conducted with 957 adults (>60 y), Laird et al. (51) showed a significant association between low vitamin D status (25-(OH)D <25 nmol/L) and inflammation markers including IL-6, TNFα, IL-10, and CRP.

After Vit-D supplementation we noted a significant decrease in the Th1/Th2 ratio in link with TNFα and IL-6 reduced levels. This is in accordance with Penna's data (52) showing that 1,25-(OH)_2_D can inhibit Th1 differentiation (via expression of IFNγ) and increase the Th2 response by stimulating IL-5 production. The IFN-γ/IL-5 ratio is of interest when evaluating the Th1/Th2 balance (53, 54).

Interestingly, the Vit-D supplementation was associated with an increase in TGFβ plasma levels after influenza vaccination, while no change in the Treg cell sub-population was observed. Likewise, previous studies showed that neuraminidase from influenza vaccine strains directly activates TGFβ production (55), which contributes to the tolerogenic effect of Vit-D on cell-mediated immunity (44). Increased IDO activity has been associated with tolerogenic immune responses (56, 57). In our study, IDO activity was not changed after Vit-D supplementation, which is consistent with an unchanged Treg sub-population.

Previous results (58) demonstrate that 1,25-(OH)_2_D strongly up-regulates the cathelicidin gene and protein expression NOX2-dependently, and induces antibacterial activity by NADPH oxidase pathway in phagocytes (19). In our conditions, we did not observe any effect on PMN ROS production after Vit-D supplementation. These conflicting findings highlight the need to characterize the role of the NOX2-dependent ROS signaling pathway in Vit-D-induced cathelicidin's anti-infectious effects.

## Conclusion

Our data demonstrate for the first time that Vit-D supplementation in deficient elderly persons promotes a higher TGFβ plasma level in response to influenza vaccination without improving antibody production. The Vit-D supplementation seems to direct the lymphocyte polarization toward a tolerogenic immune response as suggested by the lower Th1/Th2 ratio compared to controls. Taken together, our results suggest that vitamin D supplementation in deficient elderly persons is not an effective way to improve their antibody response to influenza vaccine. A deeper characterization of metabolic and molecular pathways of these observations will aid in the understanding of vitamin D's effects on cell-mediated immunity in deficient elderly persons.

## Author Contributions

NG-M, AG, BE, HL, and M-PV designed the protocol. NG-M, JT, PB, and MP-V performed the experiments, analyzed the data and wrote the manuscript. CD and VC conducted the medical visits. GM and VS participated to the biological analysis and serum vit D quantification. All authors read and approved the manuscript.

### Conflict of Interest Statement

The authors declare that the research was conducted in the absence of any commercial or financial relationships that could be construed as a potential conflict of interest.
